# Enhancement of Thermal Resistance by Metal Ions in Thermotolerant *Zymomonas mobilis* TISTR 548

**DOI:** 10.3389/fmicb.2020.00502

**Published:** 2020-03-31

**Authors:** Tomoyuki Kosaka, Aya Nishioka, Tomoko Sakurada, Kento Miura, Sakunda Anggarini, Mamoru Yamada

**Affiliations:** ^1^Graduate School of Science and Technology for Innovation, Yamaguchi University, Yamaguchi, Japan; ^2^Research Center for Thermotolerant Microbial Resources, Yamaguchi University, Yamaguchi, Japan; ^3^Faculty of Agriculture, Yamaguchi University, Yamaguchi, Japan

**Keywords:** *Zymomonas*, thermotolerance, metals, magnesium, potassium

## Abstract

The thermal resistance of fermenting microbes is a key characteristic of stable fermentation at high temperatures. Therefore, the effects of various metal ions on the growth of *Zymomonas mobilis* TISTR 548, a thermotolerant ethanologenic bacterium, at a critical high temperature (CHT) were examined. Addition of Mg^2+^ and K^+^ increased CHT by 1°C, but the effects of the addition of Mn^2+^, Ni^2+^, Co^2+^, Al^3+^, Fe^3+^, and Zn^2+^ on CHT were negligible. To understand the physiological functions associated with the addition of Mg^2+^ or K^+^, cell morphology, intracellular reactive oxygen species (ROS) level, and ethanol productivity were investigated at 39°C (i.e., above CHT). Cell elongation was repressed by Mg^2+^, but not by K^+^. Addition of both metals reduced intracellular ROS level, with only K^+^ showing the highest reduction strength, followed by both metals and only Mg^2+^. Additionally, ethanol productivity was recovered with the addition of both metals. Moreover, the addition of Mg^2+^ or K^+^ at a non-permissive temperature in 26 thermosensitive, single gene-disrupted mutants of *Z*. *mobilis* TISTR 548 revealed that several mutants showed metal ion-specific growth improvement. Remarkably, K^+^ repressed growth of two mutants. These results suggest that K^+^ and Mg^2+^ enhance cell growth at CHT via different mechanisms, which involve the maintenance of low intracellular ROS levels.

## Introduction

Recently, bioethanol has gained attention as an alternative to fossil fuel because as a carbon-neutral fuel, it can potentially delay the progress of global warming (Hahn-Hägerdal et al., 2006; Chisti, 2008). However, industrial scale bioethanol production requires a more cost-effective process to be economically competitive. High-temperature fermentation (HTF; which enables fermentation at a temperature 5–10°C higher than that used in the conventional process) may reduce (1) cooling cost, (2) running cost at the simultaneous saccharification and fermentation stage, and (3) contamination risks (Abdel-Banat et al., 2010; Kosaka et al., 2018). Bioethanol production by HTF requires high-efficiency ethanol production and thermotolerant microorganisms. *Zymomonas mobilis*, a Gram-negative, facultative, anaerobic bacterium, performs high-speed ethanol production (He et al., 2014) compared with the conventional ethanol producer *Saccharomyces cerevisiae*, whose cultivation temperature of >35°C is not permissible for yeast growth (Aldiguier et al., 2004). *Z*. *mobilis*, which uses the Entner–Doudoroff pathway and an incomplete TCA cycle, is facultatively anaerobic and requires no oxygen for its growth; conversely, it assimilates glucose, fructose, and sucrose as the sole carbon sources (Panesar et al., 2006; He et al., 2014). We focused on *Z*. *mobilis* TISTR 548, one of the thermotolerant *Z*. *mobilis* strains that grew at 39°C (Sootsuwan et al., 2007), and developed thermotolerant mutants by thermal adaptation enhancement of its critical high temperature (CHT), an upper limit for survival, up to 2°C (Matsushita et al., 2016; Kosaka et al., 2019). We subsequently used this mutant strain with HTF using a model fermentation and distillation system to reveal the effectiveness of this method and bioethanol productivity by HTF with *Z*. *mobilis* (Murata et al., 2015).

Metal ions enhance the growth of ethanologenic microorganisms at CHT. Microorganisms require several ions as essential metals for the normal function and homeostasis of a wide range of cellular proteins (Reid et al., 2009), but these ions are toxic at high concentrations (Gadd, 1992). Among these ions, only Mg^2+^ has been reported to enhance thermotolerance in *Z*. *mobilis* strains (Thanonkeo et al., 2007). Moreover, Mg^2+^ helped recover thermosensitive mutants of *Z*. *mobilis* TISTR 548, in which genes for membrane stabilization or membrane formation were disrupted, suggesting that at CHT, Mg^2+^ stabilizes membrane structure and protects cells from heat (Charoensuk et al., 2017). Mg^2+^ also stabilizes the outer membrane (OM) structure, at least of lipopolysaccharide (LPS), of cells by divalent cation crossbridging (bridging action) in Gram-positive bacteria (Nikaido, 2003). Studies on several microorganisms, particularly *Escherichia coli* (Murata et al., 2011) and *Lactobacillus* strains (Yang et al., 2017), have revealed the thermotolerance-enhancing effect of Mg^2+^. However, although the enhancing effects of K^+^ and Ca^2+^ on *S*. *cerevisiae* (Lam et al., 2014) and lactic acid bacteria (Huang and Chen, 2013), respectively, have been reported, there is no report about the enhancement effects of these and other metals on *Z*. *mobilis* growth at CHT. This evidence suggests that the concentration of several metals in a fermentation medium is important for efficient HTF for bioethanol production. However, the effect of a wide range of metals on *Z*. *mobilis* TISTR 548 at CHT is yet to be investigated. Besides, the mechanism underlying the effects of these metals, such as Mg^2+^, on cell physiology at CHT remains unclear.

In this study, the effects of metal ions, i.e., Mn^2+^, Ni^2+^, Co^2+^, Al^3+^, Fe^3+^, Zn^2+^, Mg^2+^, K^+^, and Ca^2+^, on *Z*. *mobilis* TISTR 548 growth at CHT were observed. Moreover, the effects of Mg^2+^ and K^+^ (these metals enhanced growth at CHT) on the physiology of *Z*. *mobilis* TISTR 548 and its thermosensitive mutants were investigated.

## Materials and Methods

### Bacterial Strains, Media, and Cultivation Conditions

The bacterial strains used in this study are listed in Table 1. To grow *Z*. *mobilis*, a preculture was prepared in 2 mL of YPD medium (0.3% yeast extract, 0.5% peptone, and 3% glucose) and incubated overnight at 30°C. The overnight culture was subsequently inoculated into fresh YPD medium at an OD_550_ of 0.05. Cultivation was performed under non-shaking (static) conditions.

**TABLE 1:** List of *Zymomonas mobilis* strains used in this study.

**Strain**	**Genotype**	**Reference or source**
TISTR 548		TISTR collections
TC01	TISTR 548 (*ZZ6_0707*:Tn*10*)	Charoensuk et al., 2017
TC03	TISTR 548 (*ZZ6_1376*:Tn*10*)	Charoensuk et al., 2017
TE12	TISTR 548 (*ZZ6_1146*:Tn*10*)	Charoensuk et al., 2017
C12-36	TISTR 548 (*ZZ6_1551*:Tn*10*)	Charoensuk et al., 2017
C11-44	TISTR 548 (*ZZ6_1046*:Tn*10*)	Charoensuk et al., 2017
C13-36	TISTR 548 (ZZ6_1210:Tn10)	Charoensuk et al., 2017
TC04	TISTR 548 (ZZ6_0923:Tn10)	Charoensuk et al., 2017
1-2	TISTR 548 (ZZ6_1043:Tn10)	Charoensuk et al., 2017
3-24	TISTR 548 (ZZ6_0929:Tn10)	Charoensuk et al., 2017
TC14	TISTR 548 (ZZ6_0158:Tn10)	Charoensuk et al., 2017
C31-23	TISTR 548 (ZZ6_1254:Tn10)	Charoensuk et al., 2017
TC15	TISTR 548 (ZZ6_1477:Tn10)	Charoensuk et al., 2017
F32	TISTR 548 (ZZ6_0616:Tn10)	Charoensuk et al., 2017
C12-43	TISTR 548 (ZZ6_0934:Tn10)	Charoensuk et al., 2017
TC10	TISTR 548 (ZZ6_0681:Tn10)	Charoensuk et al., 2017
C12-44	TISTR 548 (ZZ6_0023:Tn10)	Charoensuk et al., 2017
C21-17	TISTR 548 (ZZ6_1659:Tn10)	Charoensuk et al., 2017
TC05	TISTR 548 (ZZ6_0980:Tn10)	Charoensuk et al., 2017
TC12	TISTR 548 (ZZ6_0702:Tn10)	Charoensuk et al., 2017
TE19	TISTR 548 (ZZ6_0979:Tn10)	Charoensuk et al., 2017
C31-15	TISTR 548 (ZZ6_0019:Tn10)	Charoensuk et al., 2017
TC11	TISTR 548 (ZZ6_0840:Tn10)	Charoensuk et al., 2017
C12-37	TISTR 548 (ZZ6_0962:Tn10)	Charoensuk et al., 2017
TC09	TISTR 548 (ZZ6_0541:Tn10)	Charoensuk et al., 2017
TC13	TISTR 548 (ZZ6_0861:Tn10)	Charoensuk et al., 2017
1-10	TISTR 548 (ZZ6_1289:Tn10)	Charoensuk et al., 2017

### Examination of the Effects of Various Materials on Cell Growth

To compare the effects of additional reagents, cells were subjected to two-step cultivation (Kosaka et al., 2019) at the same temperature to observe the effect of temperature or additional reagents. Two-step cultivation can simply determine the temperature-upper limit for the survival of cells because when the first culture is performed at a temperature just above a CHT, cells cannot grow in the second culture at the same temperature (Kosaka et al., 2019). In the first culture, the OD value of the culture increases even at a temperature over CHT because of cell elongation. The CHT of *Z. mobilis* TISTR 548 has been determined to be 38°C by this method (Kosaka et al., 2019). Reagents were added to a medium at the desired condition before each inoculation. Briefly, the first cultivation was performed until the culture attained a late log phase at a temperature around a putative CHT; then, a portion of the first culture was transferred into a fresh medium at an OD_550_ of 0.05 and cultured at the same temperature. All metals tested were obtained in the form of chloride salts.

### Cell Morphology

Cell morphology was observed using phase-contrast microscopy (E6F-RFK-1, Nikon, Tokyo, Japan). In total, 100 cells were randomly selected on microphotographs, and their length was measured using ImageJ (Schneider et al., 2012).

### Intracellular Reactive Oxygen Species Level

*Zymomonas mobilis* TISTR 548 cells were grown on YPD medium at 39°C. At 12 h, 5 μM H_2_DCFDA was added to the first culture, and further cultivation was performed at 39°C for 30 min. Then, cells were harvested by low-speed centrifugation and washed once with phosphate-buffered saline [130 mM NaCl, 10.8 mM Na_2_HPO_4_, 4.2 mM NaH_2_PO_4_ (pH 7.2)]. The washed cells were disrupted by sonication for 30 min using an ultrasonic cell disruptor (Bioruptor; Cosmo Bio, Tokyo, Japan) and subjected to low-speed centrifugation. Supernatant fluorescence was measured using a microplate reader (POWERSCAN^®^ HT; BioTek Instruments, Inc., Winooski, VT, United States). Protein concentration was determined using the Lowry method (Dulley and Grieve, 1975). The result obtained for intracellular reactive oxygen species (ROS) levels is expressed as fluorescence intensity per protein concentration, and the ratio of the number of cells grown in the presence of a metal ion to that of cells grown in its absence was estimated and expressed as percentage.

### Ethanol Concentration

Ethanol concentration was analyzed using a gas chromatograph (GC-2014, Shimadzu, Kyoto, Japan) equipped with a flame ionization detector and Gaskuropack 54-packed glass column (60/80 mesh; GL Science, Tokyo, Japan); nitrogen was used as a carrier gas (flow rate, 35 mL/min). Operating temperatures were as follows: injection temperature, 200°C; column temperature, 180°C; and detector temperature, 200°C.

## Results

### Effects of Metal Ions on *Z*. *mobilis* TISTR 548 Growth at CHT

**FIGURE 1:**
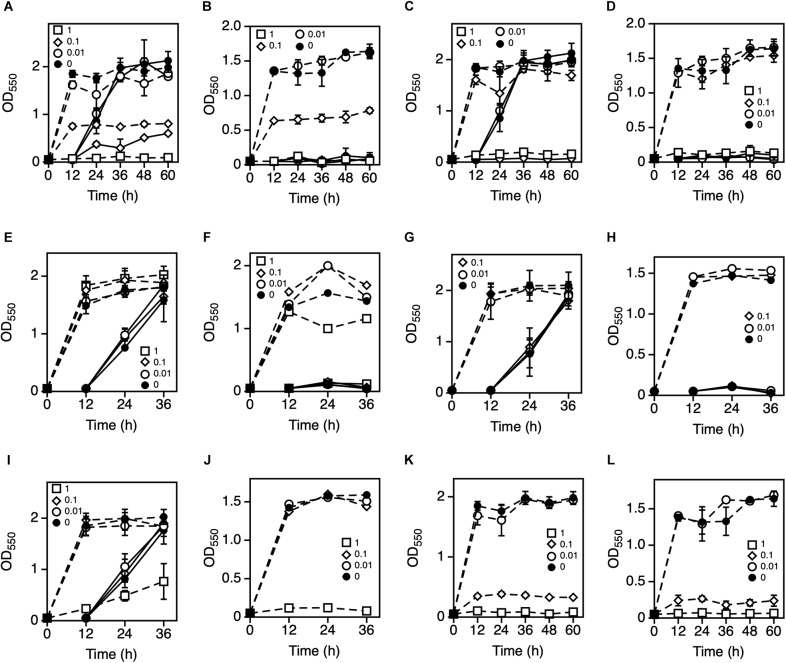
Effects of various metal ions on the two-step cultivation of *Zymomonas mobilis* TISTR 548. Cells were cultivated in YPD medium at 38°C with different concentrations of NiCl_2_ **(A)**, ZnCl_2_ **(C)**, FeCl_3_ **(E)**, AlCl_3_ **(G)**, MnCl_2_ **(I)**, or CoCl_2_ **(K)** and at 39°C with NiCl_2_ **(B)**, ZnCl_2_ **(D)**, FeCl_3_ **(F)**, AlCl_3_ **(H)**, MnCl_2_ **(J)**, or CoCl_2_ **(L)** under a static condition. These symbols indicate the means of three replicates, and error bars indicate standard deviations: closed circle, control (0 mM); open circle, 0.01 mM; open diamond, 0.1 mM; and open square, 1.0 mM. Dotted and solid lines indicate the OD values of the first and second stages of cultivation, respectively.

**FIGURE 2:**
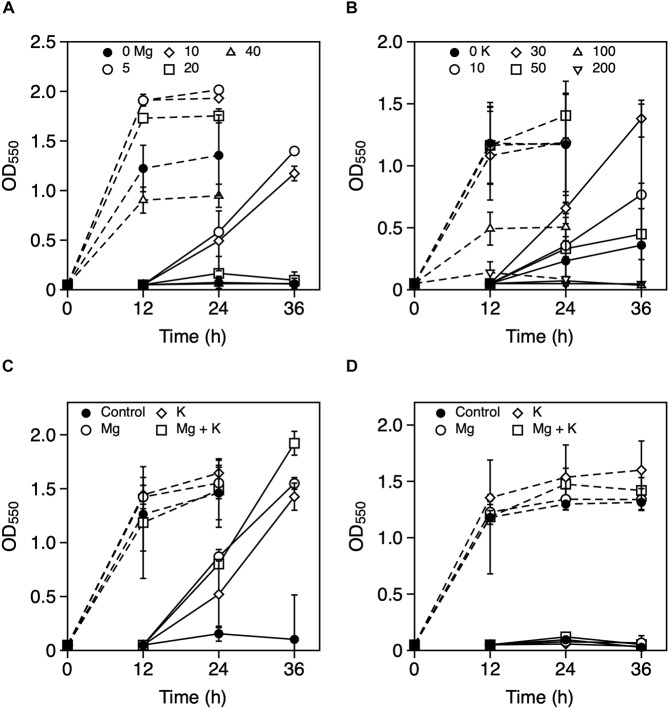
Effects of Mg^2+^ and K^+^ on the growth of *Zymomonas mobilis* TISTR 548 at critical high temperatures. Cells were cultivated in YPD medium with several concentrations of MgCl_2_ (**A:** closed circle, 0 mM; open circle, 5 mM; open diamond, 10 mM; open square, 20 mM; and open triangle, 40 mM) and KCl (**B:** closed circle, control, 0 mM; open circle, 10 mM; open diamond, 20 mM; open square, 50 mM; open triangle up, 100 mM; and open triangle down, 200 mM) at 39°C under a static condition. At 39°C **(C)** and 39.5°C **(D)**, 5 mM MgCl_2_ (open circle), 30 mM KCl (open diamond), and a combination of these metals (open square), were added, and two-step cultivation was performed. Values and error bars represent means and standard deviations, respectively, for triplicate cultures.

To explore the metal ions that enhance *Z*. *mobilis* TISTR 548 growth at putative CHT, the growth levels with and without the addition of Ni^2+^, Zn^2+^, Fe^3+^, Al^3+^, Mn^2+^, Co^2+^, Mg^2+^, and K^+^ were compared. The effect of the addition of metal ions was evaluated with two-step cultivation, wherein only viable and culturable cells grow, whereas dead or viable but non-culturable cells do not grow in fresh medium at the second cultivation (Kosaka et al., 2019). At 38°C and 39°C, the growth level under the conditions of 0.01 mM NiCl_2_, ZnCl_2_, FeCl_3_, AlCl_3_, MnCl_2_, and CoCl_2_ was the same as that without the addition of metal ions (Figure 1). On the contrary, the addition of >0.1 mM NiCl_2_ and CoCl_2_ led to a lower growth level than no addition of metal ions at the first stage of cultivation (Figures 1A,B,K,L). Similarly, the growth level following the addition of ZnCl_2_ and MnCl_2_ was lower at 1 mM (Figures 1C,D,I,J). The growth trend did not change distinctly between 38 and 39°C (Figure 1). Ten millimolar CaCl_2_ or 10 mM NaCl suppressed growth in the second step of *Z. mobilis* TISTR 548 cultivation at 38°C (data not shown). On the other hand, when MgCl_2_ and KCl were added to the medium, there was a 1° higher growth than there was without adding metals even at 39°C (Figures 2A–C). At 39.5°C, there was negligible growth in the presence of MgCl_2_ and KCl (Figure 2D). The results suggested that the optimum concentrations of MgCl_2_ and KCl for growth enhancement at 39°C were 5 and 30 mM, respectively.

### Physiological Effects of Mg^2+^ and K^+^ on *Z*. *mobilis* TISTR 548 at CHT

**FIGURE 3:**
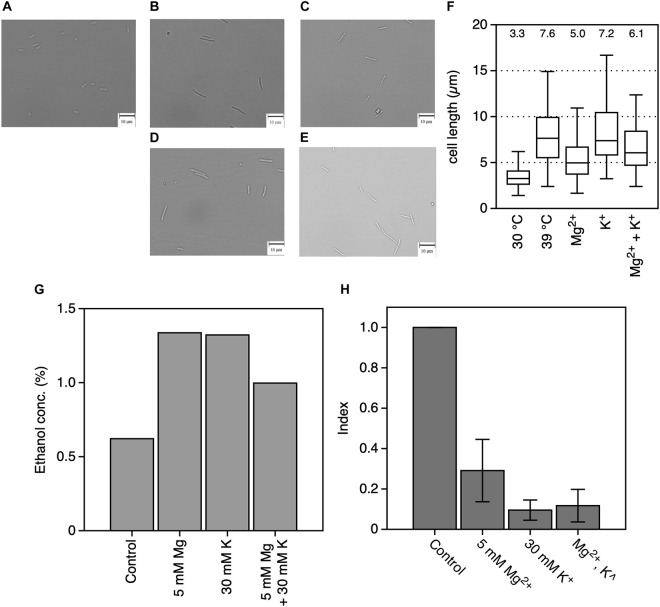
Effects of Mg^2+^ and K^+^ on the physiology of *Zymomonas mobilis* TISTR 548 at critical high temperatures. Under all conditions, the final concentrations of the added MgCl_2_ and KCl were 5 and 30 mM, respectively. **(A–E)** Morphology of cells grown in YPD medium at 30°C **(A)** or 39°C **(B)** with MgCl_2_ **(C)**, KCl **(D)**, or both **(E)** for 7 h under a static condition. Photographs were taken at a magnification of ×400. Bars indicate 10 μm. **(F)** A box plot of cell length from 100 cells measured in these conditions. The median cell length under each condition is shown as a number. **(G)** Ethanol concentration was measured in culture at 39°C for 12 h in YPD medium under a static condition. **(H)** The intracellular ROS level was measured in culture at 39°C for 12 h. Index was calculated as the ratio of the fluorescence intensity and protein concentration to the values of the control. Values and error bars represent means and standard deviations, respectively, for triplicate experiments.

Our previous report indicated that the cell length of *Z*. *mobilis* increased at CHT, and this increase reduced in thermotolerance-enhanced mutants (Kosaka et al., 2019). Indeed, cells grown at 39°C had longer cells than those grown at 30°C, which had granular shapes (Figures 3A,B). Cell morphology observed following the addition of MgCl_2_ or KCl indicated that cell length increased, with the increase in cell length being relatively lower following the addition of Mg^2+^ at 39°C than without the addition of metal ions (Figure 3C). On the other hand, the addition of KCl had no clear effect on cell length at 39°C, with a predominance of longer filamentous cells (Figure 3D). Cells cultured with both metals showed a mixture of granular and long filamentous shapes (Figure 3E). Indeed, the median value of measured cell length at 30°C, at 39°C, with MgCl_2_ at 39°C, with KCl at 39°C, or with both metals at 39°C was 3.3, 7.6, 5.0, 7.2, or 6.1 μm, respectively (Figure 3F). Ethanol productivity at 39°C was also recovered to be close to the theoretical yield by adding MgCl_2_ or KCl (Figure 3G). Accumulation of intracellular ROS was observed in *Z*. *mobilis* TISTR 548 at CHT (Kosaka et al., 2019). Addition of MgCl_2_ or KCl considerably reduced intracellular ROS levels at 39°C, and the reduction strength was the highest for only K^+^, followed by that for both metals and then only Mg^2+^ (Figure 3H).

### Effects of Mg^2+^ and K^+^ on *Z*. *mobilis* TISTR 548 Growth at CHT

**FIGURE 4:**
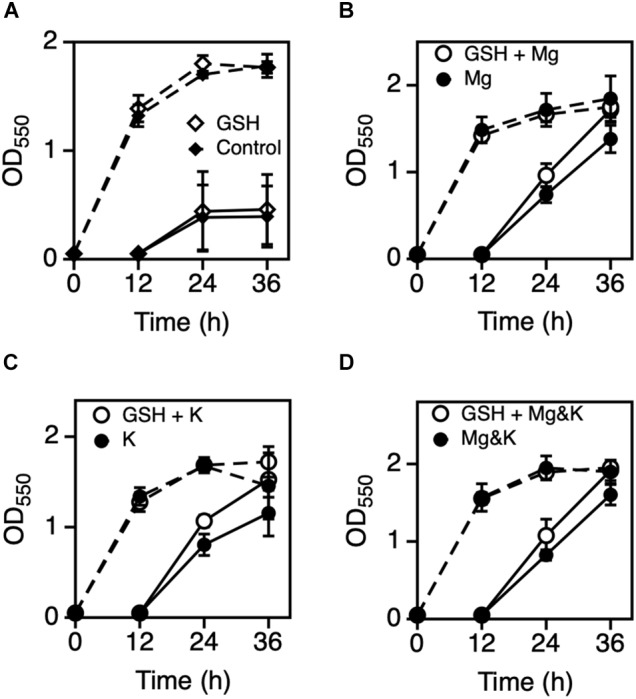
Effect of GSH with Mg^2+^ or K^+^ on the growth of *Zymomonas mobilis* TIST R548. **(A)** Cells were grown in YPD medium with (open diamond) or without 0.5 mM GSH (control, closed diamond) at 39°C under a static condition. **(B–D)** Cells were grown in YPD medium containing 5 mM MgCl_2_ **(B)**, 30 mM KCl **(C)**, or both 5 mM MgCl_2_ and 30 mM KCl **(D)** with (open circle) or without 0.5 mM GSH (closed circle) at 39°C under a static condition. After 12 h, the first-stage culture (dotted lines) was inoculated into a fresh medium and subjected to subsequent (second stage) cultivation (solid lines) under the same medium condition. Additional and non-additional conditions of GSH are shown as open and closed circles, respectively. Values and error bars represent means and standard deviations, respectively, for triplicate cultures.

**FIGURE 5:**
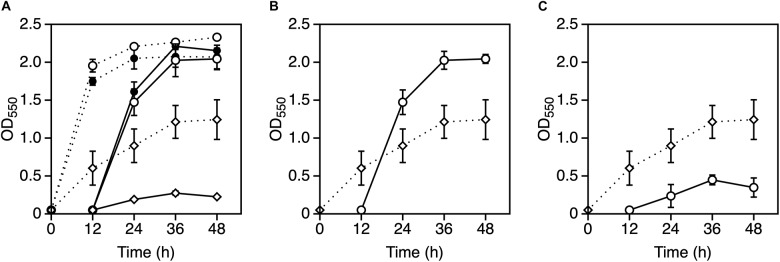
Effect of EDTA with Mg^2+^ or K^+^ on the growth of *Zymomonas mobilis* TISTR 548. **(A)** Cells were subjected to two-step cultivation (dotted line, first; solid line, second) in YPD medium with 0.01 mM EDTA (open circle), 0.05 mM EDTA (open diamond) or without EDTA (closed circle) at 38°C under a static condition. **(B,C)** At the first stage of cultivation, cells were cultured in YPD medium containing 0.05 mM EDTA (open diamond) at 38°C, and after 12 h, at the second stage of cultivation (open circle), 5 mM MgCl_2_ **(B)** or 30 mM KCl **(C)** was added to YPD medium containing 0.05 mM EDTA. Values and error bars represent means and standard deviations, respectively, for triplicate cultures.

Previous results indicated that Mg^2+^ and K^+^ somehow affect the cell physiology of *Z*. *mobilis* TISTR 548 at CHT and reduce intracellular ROS levels but probably by different mechanisms. Several bacteria use glutathione as a reducing agent to maintain a strongly reducing environment in cells, and glutathione peroxidase is an ROS-scavenging enzyme (Cabiscol Català et al., 2000). We observed the effect of glutathione with MgCl_2_ or KCl on cell growth when glutathione was added at several concentrations: 4 mM glutathione inhibited cell growth at 39°C (data not shown) but 0.5 mM did not (Figure 4A). An Mg^2+^ plus glutathione effect was observed, but the effect was not considerably distinct from that observed following the addition of K^+^ or both metals (Figures 4B–D). Next, an effective concentration of EDTA as a chelator of a divalent cation on cell growth at a CHT were explored, and then effects of metals under the presence of such a concentration of EDTA at a CHT were examined. When 0.05 mM EDTA was added to culture, cell growth was inhibited at 38°C (Figure 5A). MgCl_2_ or KCl was subsequently added under the above condition, and the resulting effect was observed. The addition of Mg^2+^ rescued EDTA inhibition at CHT (Figure 5B), but that of K^+^ did not (Figure 5C).

### Effects of Mg^2+^ and K^+^ on the Growth of Thermosensitive Mutants

In a previous study, 26 thermosensitive single gene-disrupted mutants of *Z*. *mobilis* TISTR 548 were isolated (Charoensuk et al., 2017). To observe the effects of the addition of Mg^2+^ and K^+^ on these thermosensitive mutants, their growth upon the addition of each metal was examined. Addition of Mg^2+^ significantly enhanced the growth of three mutants, whereas it inhibited the growth of one mutant (Table 2). Further, the addition of K^+^ enhanced the growth of nine mutants, i.e., almost one-third of all thermosensitive mutants, but it inhibited the growth of one mutant (C13-36) at 39.5°C (Table 2). Remarkably, the addition of both Mg^2+^ and K^+^ enhanced the growth of only one mutant with gene encoding for phospholipase D; they had the opposite effect on the mutant TC13 (Table 2). These results also suggest that Mg^2+^ and K^+^ affect the cell physiology of *Z*. *mobilis* TISTR 548 differently at CHT.

**TABLE 2:** Effects of Mg^2+^ and K^+^ on the growth of thermosensitive mutants from *Zymomonas mobilis* TISTR 548.

**Group**	**Tn10-inserted gene^a^**	**Strain**	**Function**	**Protein type**	**Growth^b^**			**Effect of MgCl_2_^c^**	**Effect of KCl^c^**
					**38°C**	**39°C**	**39.5°C**	**Ratio (%)**	**Ratio (%)**
WT (TISTR 548)					++++	++++	+++	108±10	105 ± 15
General metabolism	ZZ6_0707	TC01	Glucose sorbosone dehydrogenase	Soluble	+	+	−	114 ± 8	103 ± 23
	ZZ6_1376	TC03	5,10-methylenetetrahydrofolate reductase	Soluble	++++	++++	+	126 ± 1	118 ± 4
Membrane stabilization	ZZ6_1146	TE12	Glucosamine/fructose 6-phoshate aminotransferase	Membrane	+	+	−	119 ± 6	**328 ± 14**
	ZZ6_0929	3-24	Glycosyl transferase group 1	Soluble	+	−	−	138 ± 6	146 ± 2
	ZZ6_0923	TC04	Phospholipase D/transphosphatidylase	Membrane	−	−	−	**176 ± 7**	**177 ± 10**
	ZZ6_1551	C12-36	Squalene hopene cyclase (Shc)	Soluble	−	−	−	131 ± 4	98 ± 13
	ZZ6_1046	C11-44	Tol/Pal system component TolQ	Membrane	+	+	−	157 ± 5	**519 ± 4**
	ZZ6_1043	1-2	Tol/Pal system component TolB	Soluble	+	+	+	115 ± 9	110 ± 5
	ZZ6_1254	C31-23	Protein export membrane protein SecD	Membrane	−	−	−	112 ± 5	**223 ± 7**
	ZZ6_1477	TC15	Preprotein translocase subunit Tim44	Membrane	−	−	−	151 ± 11	127 ± 2
	ZZ6_0158	TC14	Autotransporter secretion inner membrane protein TamB	Membrane	+	−	−	118 ± 9	**165 ± 10**
	ZZ6_1210	C13-36	Competence protein ComEC	Membrane	−	−	−	126 ± 7	**33 ± 4**
	ZZ6_0840	TC11	Hypothetical transmembrane protein	Membrane	−	−	−	**181 ± 8**	126 ± 4
	ZZ6_0541	TC09	Hypothetical transmembrane protein	Membrane	++++	+++	+	119 ± 1	116 ± 5
Transporter	ZZ6_1289	1-10	Putative Fe^2+^/Mn^2+^ transporter	Membrane	−	−	−	**231 ± 3**	151 ± 4
DNA repair	ZZ6_0616	F32	DNA repair protein RadC	Soluble	++++	+++	+	113 ± 9	96 ± 4
	ZZ6_0934	C12-43	Exonuclease VII (XseA)	Soluble	−	−	−	156 ± 6	146 ± 1
	ZZ6_0681	TC10	DNA repair protein RadA	Soluble	+	+	−	101 ± 5	62 ± 8
tRNA/rRNA modification	ZZ6_0023	C12-44	tRNA/rRNA methyltransferase (SpoU)	Soluble	+++	++	++	117 ± 11	116 ± 12
Protein quality control	ZZ6_1659	C21-17	Zn-dependent peptidase	Soluble	++++	+++	++	99 ± 10	104 ± 16
	ZZ6_0980	TC05	Serine protease DegP	Soluble	−	−	−	153 ± 5	**172 ± 3**
Translational regulation	ZZ6_0702	TC12	ATP-dependent helicase HrpB	Soluble	−	−	−	98 ± 1	**212 ± 4**
Cell division	ZZ6_0979	TE19	ParA/MinD-like ATPase	Soluble	−	−	−	114 ± 8	90 ± 11
Transcriptional regulation	ZZ6_0019	C31-15	Trp repressor-binding protein WrbA	Soluble	−	−	−	137 ± 4	**577 ± 8**
Others	ZZ6_0962	C12-37	Pseudogene		+	+	−	161 ± 9	121 ± 7
	ZZ6_0861	TC13	Hypothetical protein	Soluble	+	+	−	**53 ± 3**	**327 ± 2**

*^a^Thirty-six thermotolerant genes were identified, and they were classified into 10 groups (Charoensuk et al., 2017). ^b^The growth of these mutants at 38, 39, and 39.5°C compared with that of the parental strain on YPD medium. The symbols “+” represent the degree of cell growth of mutants at a critical high temperature compared with that of the parental strain, whereas “−” indicates no growth. ^c^The ratio (%) was calculated from the OD_550_ values for cells grown at 39.5°C for 24 h with 5 mM MgCl_2_ or 30 mM KCl divided by those cells grown without metals. Values represent means ± standard deviations of three replicates. Bold values indicate a ratio of >1.5 or <0.5 against the parental strain.*

## Discussion

We observed the additional effects of Ni^2+^, Zn^2+^, Fe^3+^, Al^3+^, Mn^2+^, and Co^2+^ on *Z*. *mobilis* TISTR 548 growth at high temperatures, but these metals showed only negative effects (Figure 1). Among these, the effects of Fe^3+^ and Al^3+^ were negligible under the tested conditions (Figures 1E–H). However, 1.0 mM Ni^2+^, Zn^2+^, Mn^2+^, and Co^2+^ clearly inhibited *Z*. *mobilis* TISTR 548 growth (Figure 1). A previous report indicated that the addition of 0.35 mM Zn^2+^ markedly inhibited ethanol productivity in *Z*. *mobilis* ZM4 probably by inhibiting metabolic enzymes (Liu et al., 2010). In the case of a different microorganism, i.e., *S*. *cerevisiae*, the quantity of Zn^2+^, Mg^2+^, and Mn^2+^ required for effective fermentation was 0.01, 0.05, and 0.04 g/L, respectively (Deesuth et al., 2012), implying that 0.1 mM metals are usually required for growth, but excess concentrations can inhibit growth. This implies that only specific metals enhance the growth of specific microorganisms.

Only Mg^2+^ and K^+^ enhanced *Z*. *mobilis* TISTR 548 growth and improved CHT from 38 to 39°C (Figure 2A). At CHT, the intracellular molecular components of bacterial cells leaked (Haight and Morita, 1966; Allwood and Russell, 1967). In *S*. *cerevisiae* and probably other microorganisms, the addition of K^+^ prevents ion leakage (Lam et al., 2014). K^+^ channels are activated when tension in the lipid bilayer is increased (Iwamoto and Oiki, 2018), and Mg^2+^ transporters are induced by heat treatment in *Salmonella enterica* (O’Connor et al., 2009). Regarding quantity, these two metal ions (K^+^ and Mg^2+^) are the principal and second highest ions in bacterial cells found at concentrations of 100–500 mM (Ballal et al., 2007) and ∼1 mM (Groisman et al., 2013), respectively. Therefore, the optimal concentrations of 30 mM K^+^ and 5 mM Mg^2+^ (Figures 2A,B) are probably related to their intracellular concentrations, further suggesting that similar ion conditions enhance cell metabolism by preventing ion leakage from cells or supporting ion transportation from the extracellular space. However, the effects of Mg^2+^ and K^+^ on the two common characteristics of bacteria, namely, cell elongation and ROS accumulation, observed in *Z*. *mobilis* TISTR 548 at CHT (Matsushita et al., 2016) were different. Cell length at CHT was suppressed by the addition of Mg^2+^ but not by that of K^+^ (Figure 3F). Although ROS accumulation reduced by the addition of both metals, the addition of K^+^ showed a stronger effect than that of Mg^2+^ (Figure 3H). The GSH results indicated that the additive effect of GSH was observed in both cases of Mg^2+^ and K^+^ (Figures 4B,C), suggesting that the growth enhancement effect of Mg^2+^ or K^+^ does not arise directly from the action of GSH added exogenously. In a Gram-negative bacterium, *E. coli*, GSH is important for periplasmic redox homeostasis (Pittman et al., 2005) and heterogeneous expression of glutathione reductase allows the microbe to be hydrogen peroxide tolerance (Kim et al., 2009). It is assumed that, in *Z. mobilis* TISTR 548, GSH keeps periplasmic redox homeostasis and/or somehow makes cells tolerate oxidative stress by its reducing power, but the major effects at CHT by Mg^2+^ and K^+^ are not likely the action by GSH. Moreover, EDTA treatment showed that K^+^ did not complement the EDTA effect at CHT (Figure 5C). These results suggest that Mg^2+^ and K^+^ affect the cell physiology of *Z*. *mobilis* TISTR 548 at CHT using different mechanisms.

The effect of Mg^2+^ on the cell physiology of *Z*. *mobilis* TISTR 548 at CHT has been described: Mg^2+^ probably stabilizes membrane structure as proposed in *E*. *coli* (Charoensuk et al., 2017). Mg^2+^ stabilizes OM (Nikaido and Vaara, 1985), particularly LPS, where Mg^2+^ bridges lipid A (Nikaido, 2003). The present study results also showed that the addition of Mg^2+^ repressed cell elongation at CHT (Figure 3F) and restored the growth of the disrupted genes of *ZZ6_0923*, which encodes the cardiolipin biosynthesis protein (Table 2). However, Mg^2+^ has been thought to stabilize proteins, enhance protein–nucleic acid interactions, mitigate oxidative stress, and act as a metabolic signal (O’Connor et al., 2009). Mg^2+^ is required to maintain cell metabolism, DNA replication, transcription and translation, and DNA stabilization (Xu et al., 2018), and it plays a role in enzyme activations. For instance, Mg^2+^ stabilizes pyruvate decarboxylase, an enzyme responsible for the decarboxylation of pyruvate in central metabolism, with thiamine diphosphate serving as a cofactor (Pohl et al., 1994). Besides, phosphoglycerate kinase uses Mg^2+^ as a cofactor (Andreini et al., 2008). Addition of Mg^2+^ reduced ROS accumulation at CHT (Figure 3H), and the growth of the disrupted Fe^2+^/Mn^2+^ transporter (ZZ6_1289) recovered greatly (Table 2). Therefore, maintaining an intracellular Mg^2+^ concentration may enable heat tolerance either by ions or cytoplasmic Mg^2+^ sensors, proteins, and RNAs. (Groisman et al., 2013).

K^+^, the most dominant intracellular cation, greatly contributes to pH homeostasis and turgor maintenance as well as bacterial osmotic adaptation, pH regulation, gene expression, and cell enzyme activation (Epstein, 2003; Ballal et al., 2007). Indeed, the addition of K^+^ affected most cell physiology of *Z*. *mobilis* TISTR 548 at CHT (Table 2), e.g., the growth of 35% (9/26 strains) of mutants recovered greatly. These effects may contribute to reducing intracellular ROS levels (Figure 3H). Between these, K^+^ probably facilitates the functioning of periplasmic proteins in *Z*. *mobilis* TISTR 548 due to the growth recovery of disrupted *tolQ*, *secD*, *tamB*, and *degP* (Table 2). The amount of intracellular K^+^ directly affects membrane potential (Bakker and Mangerich, 1981), which is required for protein secretion to periplasm (Daniels et al., 1981). The membrane potential is hypothetically required for potassium transport from extracellular space to the cytoplasm via the membrane potential-driven K^+^ uptake system (Kup, ZMO1209, and ZZ6_0125) in *Z*. *mobilis*. Therefore, K^+^ may also facilitate membrane potential maintenance in *Z*. *mobilis* TISTR 548.

**FIGURE 6:**
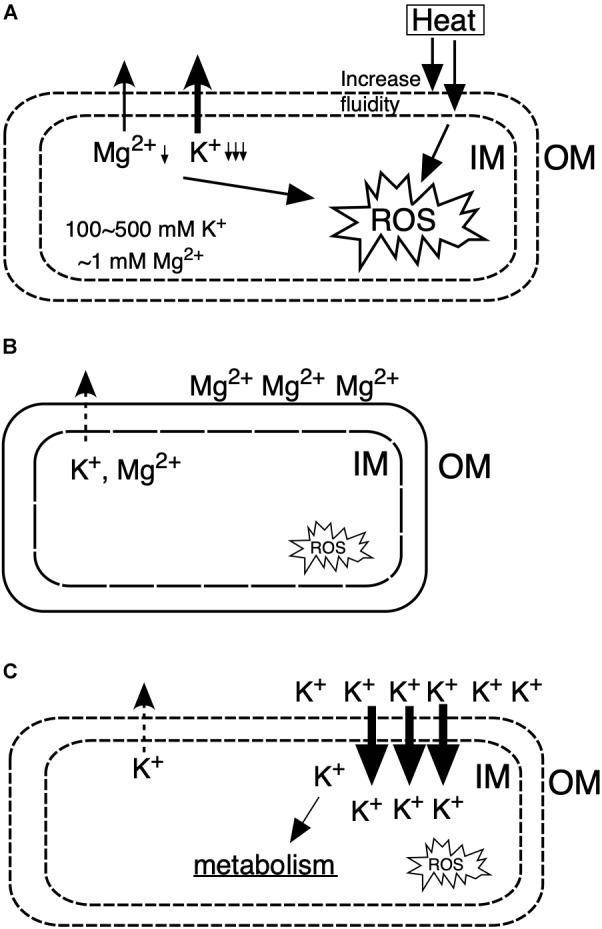
Models for the action mechanisms of Mg^2+^ and K^+^ on *Z. mobilis* TISTR 548 at CHT. **(A)** Under CHT conditions, **(B)** by the addition of Mg^2+^, **(C)** by the addition of K^+^. IM, inner membrane; OM, outer membrane; ROS, reactive oxygen species.

Under CHT conditions (Figure 6A), the inner membrane fluidity increases to cause leakages of ions from cytoplasm and electrons from the respiratory chain, which lead to the accumulation of intracellular reactive oxygen species, resulting in damage of macromolecules of DNA, RNA, proteins and lipids, and thereby cells are elongated and unable to maintain intracellular homeostasis, causing cell death. However, by the addition of Mg^2+^ (Figure 6B), the OM is stabilized by binding of Mg^2+^ and the inner membrane is also stabilized, resulting in suppression of the leakage of intracellular ions as well as the leakage of electrons from the respiratory chain. On the other hand, by the addition of K^+^ (Figure 6C), K^+^ leakage is repressed to maintain homeostasis for cellular metabolism, by which intracellular ROS is reduced. Moreover, these observations suggest that Mg^2+^ and K^+^ exhibit diverse, rather than single, effects on *Z*. *mobilis* TISTR 548. Interestingly, when both Mg^2+^ and K^+^ exist in the medium at high concentrations, their crosstalk effects on cell physiology sometimes occur. These effects are partly specific to each ion; their additive effect on cell growth at CHT was observed, but that did not entail the whole sum of their effects (Figure 2C). The thermotolerance acquisition mechanisms of *Z*. *mobilis* upon the addition of Mg^2+^ and K^+^ are more complex than the accumulated effects of their metals in accomplishing enhanced *Z*. *mobilis* growth at CHT.

## Conclusion

Among various metals, only Mg^2+^ and K^+^ enhanced the thermotolerance of *Z*. *mobilis* TISTR 548. The primary effects of Mg^2+^ and K^+^ on the cell physiology of *Z*. *mobilis* TISTR 548 are largely different, but these metals reduce intracellular ROS accumulation. Based on the study results, several strategies for improving the CHT of *Z*. *mobilis* by membrane stabilization and intracellular metabolism maintenance can be expected. Further research is needed to reveal these mechanisms for improving its growth at CHT.

## Data Availability Statement

All datasets generated for this study are included in the article/supplementary material.

## Author Contributions

AN, TS, KM, and SA conducted the experiments. TK, AN, TS, KM, and MY analyzed the data. TK, AN, and MY wrote the manuscript. All authors conceived this study.

## Conflict of Interest

The authors declare that the research was conducted in the absence of any commercial or financial relationships that could be construed as a potential conflict of interest.
